# Identification of molecular pathways and candidate genes associated with cocks’ comb size trait by genome-wide transcriptome analysis

**DOI:** 10.1038/s41598-018-20373-6

**Published:** 2018-01-31

**Authors:** Yifan Liu, Yunjie Tu, Ming Zhang, Gaige Ji, Kun Wang, Yanju Shan, Xiaojun Ju, Di Zhang, Jingting Shu, Jianmin Zou

**Affiliations:** Key Laboratory for Poultry Genetics and Breeding of Jiangsu Province, Institute of Poultry Science, Chinese Academy of Agricultural Sciences, Yangzhou, Jiangsu China

## Abstract

The comb of the male is an important secondary sexual characteristic. Although quantitative trait loci (QTLs) related to comb size have been identified, molecular mechanisms underlying this trait remain mostly unknown. In this study, RNA sequencing (RNA-seq) was employed to compare whole transcriptomic differences between two groups of Partridge Shank chickens that are divergent in comb sizes. A total of 563 differentially expressed genes (DEGs) were identified, including 277 up-regulated and 286 down-regulated DEGs. According to the animal QTL database, eight DEGs including *BMP2* and *CHADL* matching the reported QTLs were associated with the comb size. Functional annotation analysis revealed that DEGs were involved in cell communication and calcium signaling. Protein-protein interaction network analysis showed that *STK32A*, *PIK3R1*, *EDN1*, *HSPA5*, and *HSPA8* have an impact on comb growth. Moreover, potential alternative splicing events and single nucleotide polymorphisms were also identified. Our data provide a source for identifying genes and pathways with functions critical to comb size and accelerate studies involving molecular mechanisms of this sexual ornament.

## Introduction

Ornamental traits including the comb, and color of the feather and skin are important in capturing first impressions by customers in making purchase decisions. As the only observable ornamental trait in both live and slaughtered chickens, the comb is the more widely used ornamental indicator for selection^1^. The comb is a secondary sexual characteristic, and its link with the sexual maturity has been well studied^2,3^. Also, the comb influences mating decisions^4^, social ranking^5^, body temperature regulation^6^ and is associated with reproductive performance and bone mass^7^.

Comb size is a complex trait affected by many factors, including hormone concentration^8,9^ and light schedule^10,11^. Capons with significantly reduced testosterone levels have small combs^12^. High heritability of comb size (0.76) implies that this trait is not only affected by management, but is also influenced by an underlying genetic factor^13^. Discovering biomarkers including candidate genes and quantitative trait loci (QTLs) for marker-assisted selection (MAS) would accelerate genetic improvement of this trait.

To date, 530 chicken QTLs associated with comb size have been documented in the animal QTL database^14^. In the fine mapping work of comb QTLs conducted by Johnsson *et al*., candidate genes *HAO1* and *BMP2* had a potential role in comb growth and bone mass^15^. Several candidate genes, such as *VPS36*, *AR*, and *WNT11B*, have been identified as associated with comb traits^16^. However, knowledge of the genetic factors underlying comb size variations remains limited.

RNA-sequencing (RNA-seq) has been widely used in discovering transcriptomic differences in a variety of tissues related to economic traits of chickens^17–19^. To identify candidate genes and key pathways that influence comb growth in chickens, we performed a comparative analysis on the whole transcriptomes from the comb tissues of Partridge Shank roosters with distinct comb sizes using the RNA-seq technology. Our findings provided further understanding of the underlying mechanisms implicated in comb growth and would contribute to more efficient chickens breeding.

## Results

### Summary of RNA-sequencing data

In this study, six cDNA libraries were constructed using total comb RNA from three roosters with relatively bigger comb sizes (BC) and three with smaller comb sizes (SC). After quality control of the raw reads, high-quality sequence data of 43.2 gigabases (Gb) were obtained. When the data were mapped to the reference genome (*Galgal 5.0*), the ratio of the mapped reads was greater than 85.6% for each library. Detailed information on data quality and mapping statistics are presented in Supplementary Table S1.

### Identification of novel transcripts and refinement of gene structures

In total, 3,169 novel transcripts were predicted for six cDNA sequencing libraries using the Cufflinks software^20^ (Supplementary Table S2). After the protein coding potential of novel transcripts was predicted by the CPC method^21^, we found that 1,204 were protein-coding and 1,965 were non-coding. To optimize the gene annotation information in the current database, the 5′ and 3′ boundaries of known genes were refined by aligning known transcripts with the reconstructed transcripts obtained from the sequencing data. A total of 1,163 genes were refined in this manner, including 1,050 at the 5′ region and 113 at the 3′ region. The detailed annotation information regarding the structurally refined genes is provided in Supplementary Table S3.

### Alternative splicing analysis and SNP identification

**Figure 1:**
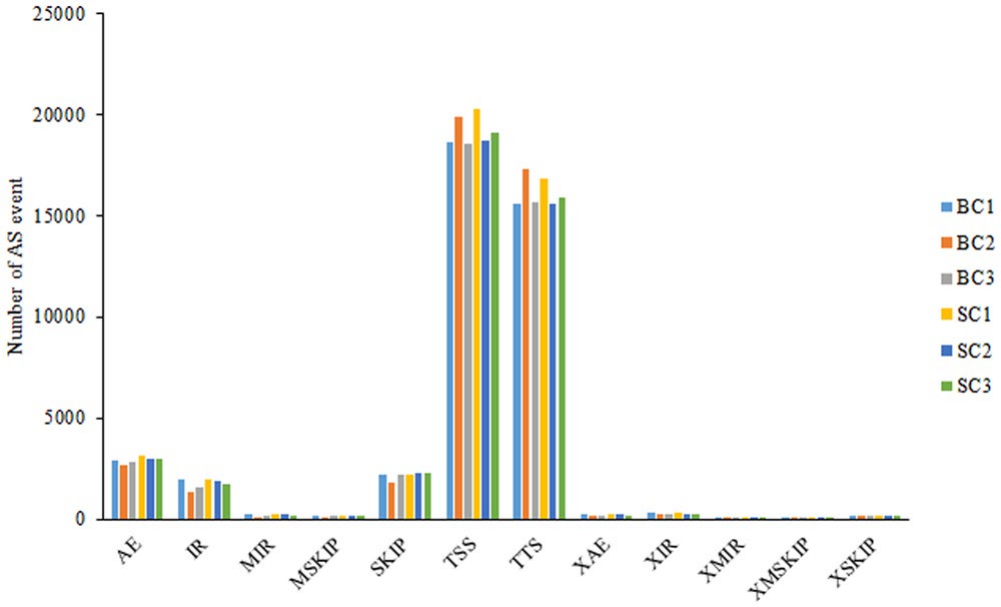
Distribution of 12 types of alternative splicing (AS) events in six samples. The six bars of different colors indicate the numbers of AS events identified in the big comb size (BC) and small comb size (SC) libraries.

From the same gene, alternative splicing (AS) could generate multiple transcripts, some of which might perform contrasting functions. We used the ASprofile software^22^ to analyze AS events in each library. Among all the 12 detected types of AS events, alternative first exons (TSS) and last exons (TTS) were the most common, accounting for more than 44% and 47%, respectively (Fig. 1 and Supplementary Table S4).

**Figure 2:**
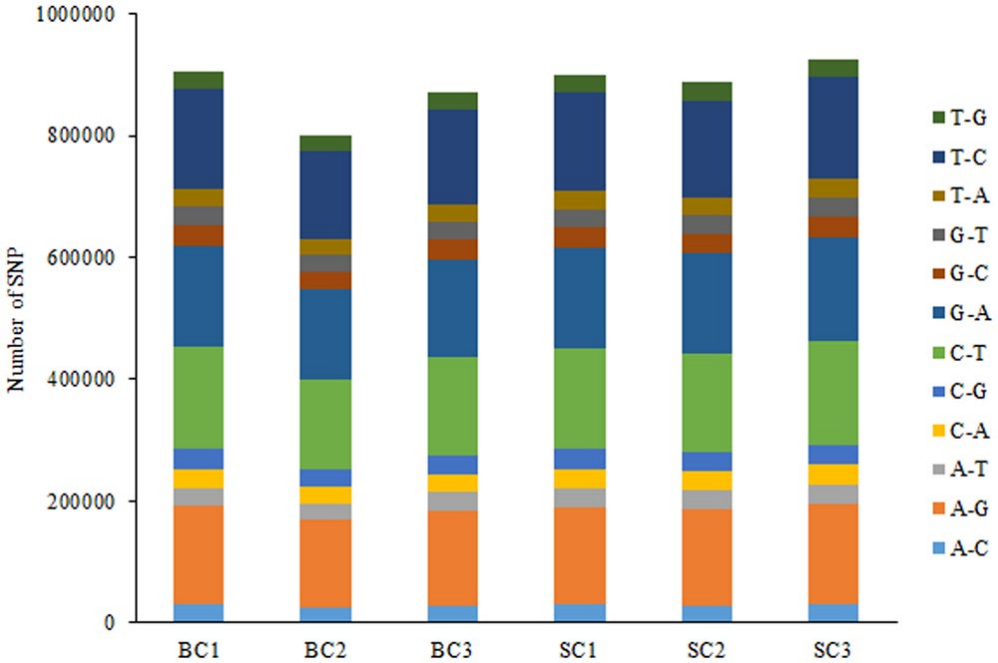
Distribution of SNPs identified in six samples. The lengths of the bars indicate the numbers of SNPs identified in the big comb size (BC) and small comb size (SC) libraries.

A total of 124,315 putative SNPs were found in all six libraries using the GATK software^23^ (Supplementary Table S5). Among them, 77.28% were located in the non-coding region, while 22.72% were in the coding region. As shown in Fig. 2, the frequencies of G/A, C/T, T/C, and A/G were by far the most.

### Gene expression analysis and identification of differentially expressed gene**s**

Expression levels of all genes were calculated using the HTSeq software^24^ and described by the reads per kilobase per million reads (RPKM). A total of 19,087 genes were detected in the six cDNA libraries, and the number of expressed genes was similar in each library (14,689~15,112). Most of the identified genes (11,381, 59.6%) had the expression levels of RPKM 1~100, whereas only a few (119, 0.6%) had expression levels of RPKM greater than 500.

**Figure 3:**
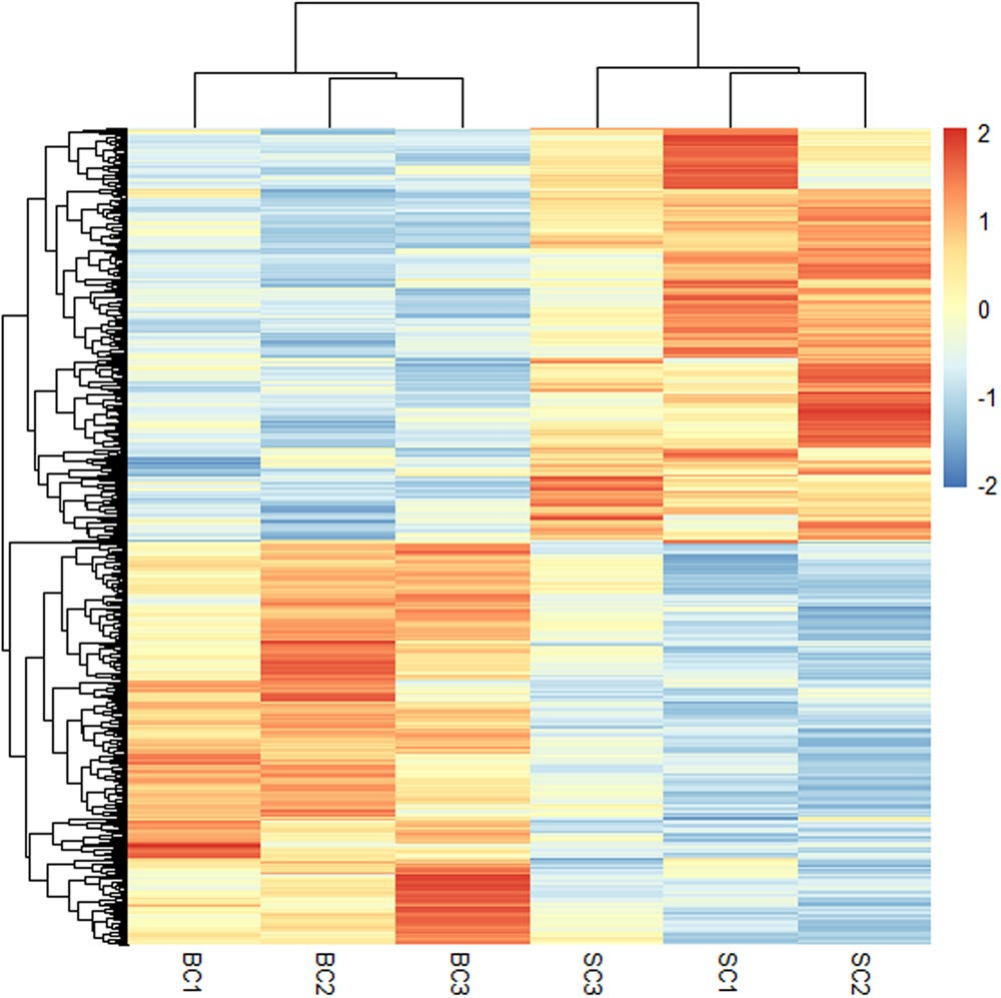
Hierarchical clustering of the differentially expressed genes between the big comb size (BC) and small comb size (SC) groups. Each column represents a sample, and each row represents a gene. The red and blue gradients indicate an increase and a decrease in gene expression abundance, respectively.

The edgeR R package^25^ was used to screen for differentially expressed genes (DEGs) between BC and SC chickens. By taking a *P*-value < 0.05 and |foldchange| > 1.5 as the cutoff, a total of 563 DEGs were identified (Fig. 3 and Supplementary Table S6). Among these DEGs, 286 genes were down-regulated and 277 were up-regulated in the BC group. Moreover, eight DEGs identified were also located in the reported comb size QTL regions, which were *BMP2*, *CHADL*, *EDA2R*, *EMP1*, *TLR5*, *FBLN1*, *PLP1*, and *HAL*.

### Quantitative real-time PCR validation

**Figure 4:**
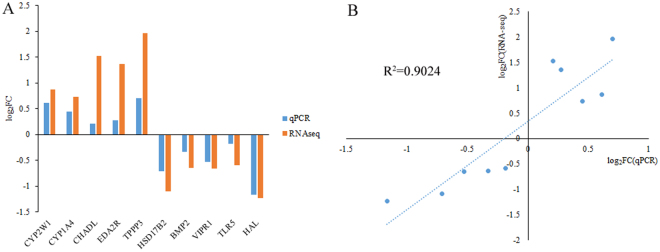
Illustrating of the qPCR confirmation results for the 10 selected differentially expressed genes. (**A**) The X-axis represents the selected 10 genes and the Y-axis represents the log_2_(foldchange) values derived from RNA-seq and qPCR. (**B**) Regression analysis of the log_2_(foldchange) values between RNA-seq and qPCR.

To validate the RNA-seq results, 10 DEGs including *CYP2W1*, *CYP1A4*, *CHADL*, *EDA2R*, *TPPP3*, *HSD17B2*, *BMP2*, *VIPR1*, *TLR5*, and *HAL* were randomly selected for quantitative real-time PCR (qPCR). As shown in Fig. 4A, all selected DEGs showed concordant expression patterns between the RNA-seq and qPCR results. There was a high positive correlation (R^2^ = 0.9024) between the computational and experimental fold changes (Fig. 4B).

### Function enrichment of differentially expressed genes

**Figure 5:**
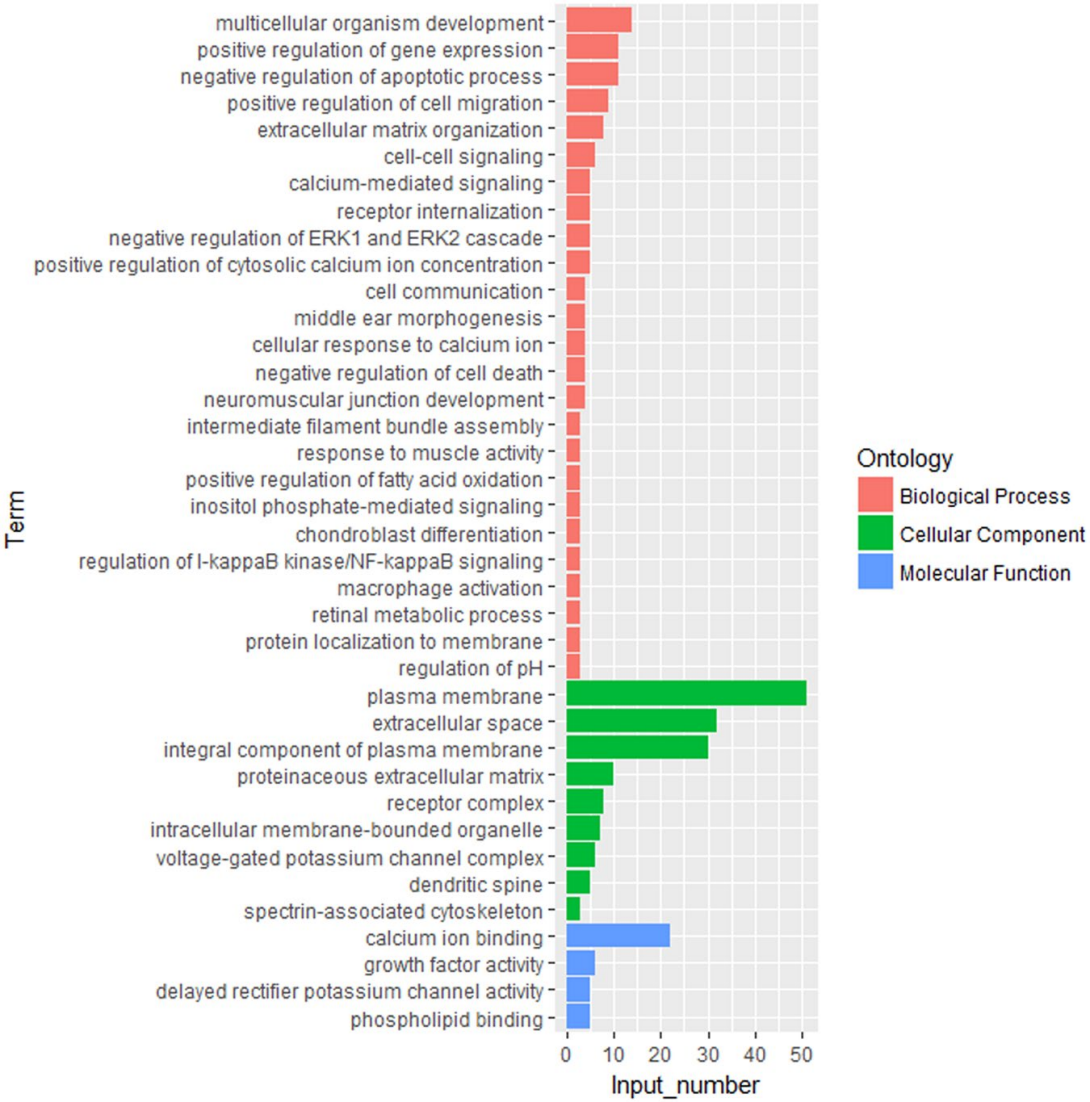
GO analysis of the differentially expressed genes between the big comb size (BC) and small comb size (SC) groups. Significantly enriched GO terms of three types are used (*P*-value ≤ 0.05). The lengths of the bars indicate the corresponding numbers of genes for each GO term.

To further elucidate the functional roles of DEGs on comb size, we performed Gene Ontology (GO) and Kyoto Encyclopedia of Genes and Genomes (KEGG) pathway enrichment analysis for the DEGs using DAVID online tool^26^. As shown in Fig. 5, there were respectively 25, 9, and 4 GO terms significantly enriched (*P*-value < 0.05) in the biological process, cellular components and molecular function. Of these enriched GO terms, four terms related to calcium ions, and multiple terms associated with the development, cell communication, and potassium channel activity were identified.

**Figure 6:**
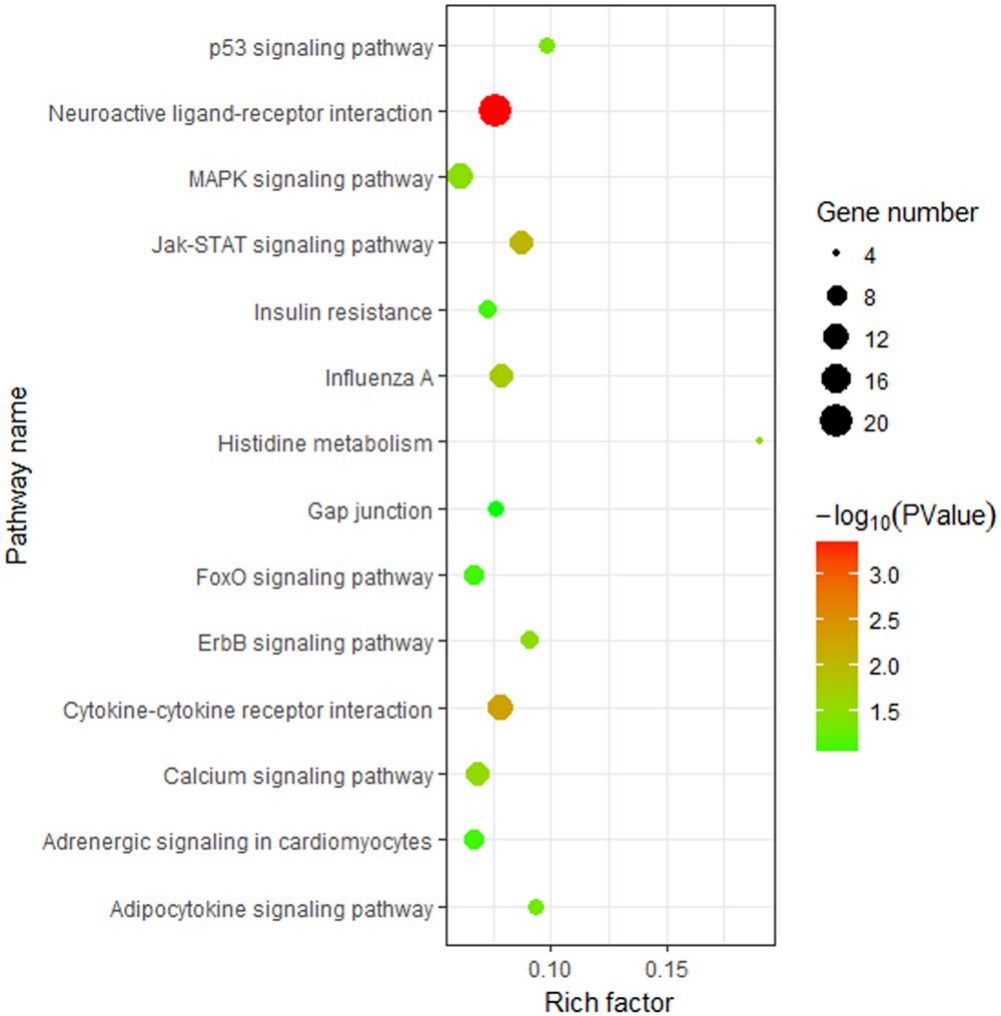
KEGG pathway analysis of the differentially expressed genes (DEGs) between the big comb size (BC) and small comb size (SC) groups. Rich factor = Amount of DEGs enriched in the pathway/Amount of all genes in the background gene set. The size and color of each bubble represent the amount of DEGs enriched in the pathway and enrichment significance, respectively.

The KEGG pathway analysis revealed 10 overrepresented pathways (*P*-value < 0.05), including neuroactive ligand-receptor interaction, cytokine-cytokine receptor interaction, Jak-STAT signaling, and calcium signaling pathway (Fig. 6). Detailed information about the enriched GO terms and KEGG pathways is provided in Supplementary Table S7.

### Protein-protein interaction analysis

**Figure 7:**
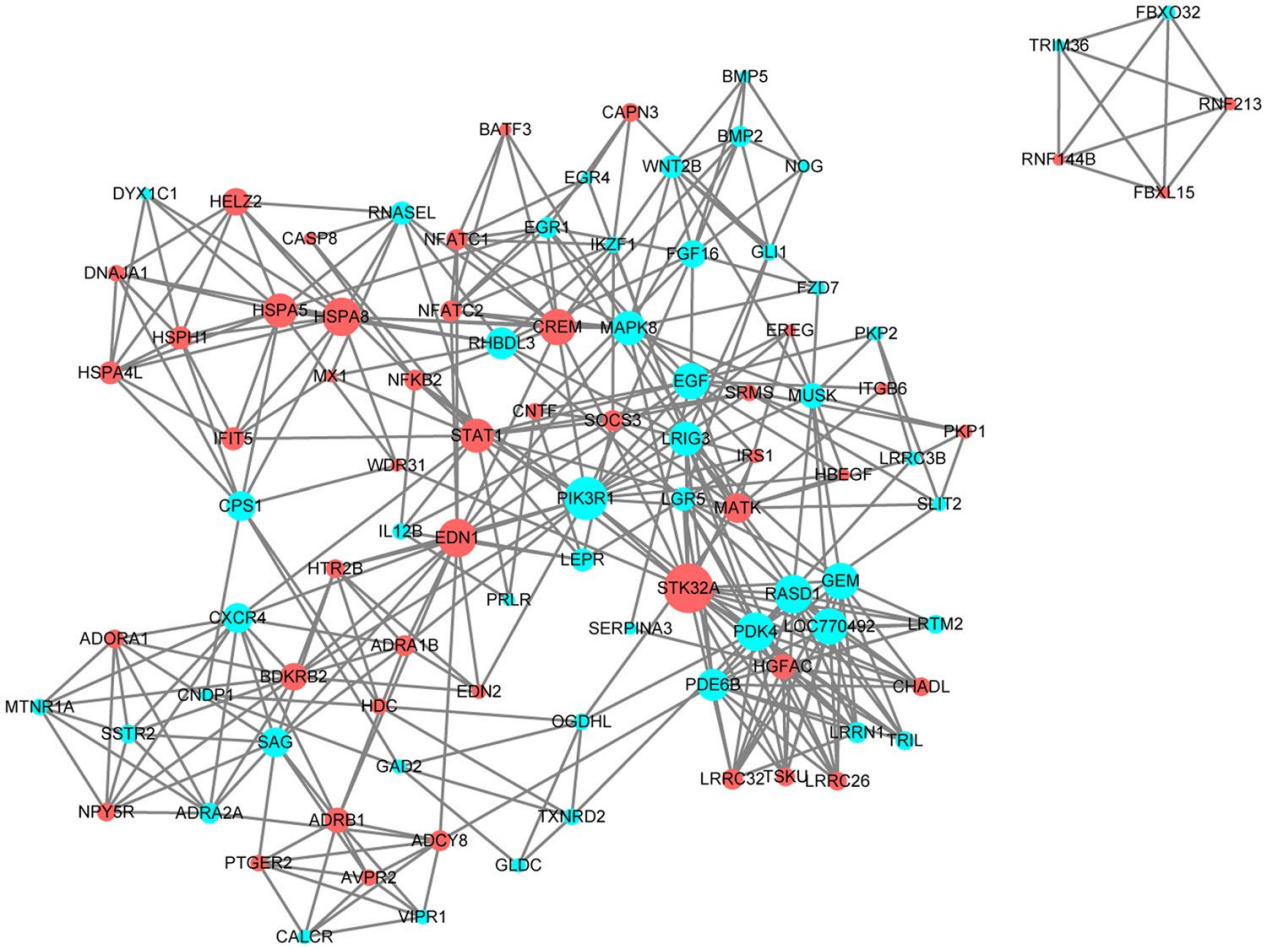
Protein-protein interaction network for the differentially expressed genes (DEGs). Node represents protein, edge represents interaction between proteins. The size of a node is proportional to degree of this node (degree of the node defined as the amount of proteins that interact with this node), and the color of the node represents the expression regulation type of DEGs (Red means up-regulation and blue means down-regulation in the BC group).

We used STRING database^27^ to predict protein-protein interactions of the detected DEGs. As shown in Fig. 7, *STK32A* and *PIK3R1* were associated with the most genes (21 and 18, respectively) that they were in the center of the interaction network. Some genes with significant variation were also identified, such as up-regulated genes *EDN1*, *HSPA5*, and *HSPA8* and down-regulated ones *EGF*, *WNT2B*, and *BMP2*. These genes were located in critical positions of the interaction network.

## Discussion

Chicken comb size has received increasing attention over the past decades, particularly in the chicken-breeding field. Although several QTLs associated with comb size have been identified, exploration at the transcriptional level is required for further refining. The Partridge Shank chicken is a Chinese local breed that mature at a young age^28^. In this study, the comb sizes of 9-week-old roosters were vastly different since the largest bird had a comb size over 4 times greater than the smallest one, even though all were reared in similar conditions. To elucidate the genetic architecture underlying comb growth, we conducted transcriptome profiling based on six Partridge Shank chickens with extraordinary comb sizes.

In this work, the newly assembled chicken genome (*Galgal* 5.0) was used for read mapping. The percentages of the mapped reads obtained per individual chicken (85.63~87.43%) were higher than those in the previous comb transcriptome study^29^ using *Galgal* 4.0 (76~78%), indicating that our results were more effective.

The rapid development of high-throughput sequencing technologies helps us understand the expression profiling of the whole genome at the transcriptional level. Compared to microarray methods, RNA-seq could provide more valuable information such as AS events and SNPs. In the present study, we identified many AS events and potential SNPs for chicken comb tissues from the SC and BC libraries. AS events and SNPs that occurred in most tissues have significant impacts on economic traits in domestic animals^30–32^. These facts implied that the AS events and SNPs identified in the current study might play an important role in comb development, though they need further investigation.

Few studies have investigated how the comb transcriptome influences comb growth in chickens. In the current research, a total of 563 DEGs and several significantly enriched pathways were identified, indicating their roles in comb size regulation. The RNA-seq results were validated by qPCR analysis, which showed a high correction rate with earlier methods. Compared to the RNA-seq results, DEGs selected for qPCR showed lower changes inexpression level, which may be explained by the different sensitivities of each method and size of test groups. Compared with the previously reported QTLs associated with comb traits, eight DEGs were highlighted, including *BMP2*, *CHADL*, *EDA2R*, *EMP1*, *TLR5*, *FBLN1*, *PLP1*, and *HAL*. Bone morphogenetic protein 2 (*BMP2*) is known for its roles in bone physiology and deposition^33,34^. Cartilage is a precursor to all bone formation. Hyaluronan is the main content of a chicken comb^35^, which is significant in cartilage metabolism^36^. In the present study, the *BMP2* expression level was down-regulated in comb tissues from the BC chickens, indicating its role in delaying comb growth by hindering cartilage metabolism. Previous works also suggest that comb growth is closely related to bone deposition, and the identified *BMP2* influences both comb growth and bone deposition as a candidate gene^15^. Another gene chondroadherin-like (*CHADL*) is expressed in cartilage and can modulate chondrocyte differentiation^37^. The expression level of *CHADL* was higher in the BC than the SC group, suggesting that this gene has a positive role in comb growth by mitigating cartilage metabolism. These results were confirmed by qPCR. Although the actual mechanism of the cartilage’s role in comb growth still remains unclear, *BMP2* and *CHADL* are suspected to be candidate genes for comb growth.

The four calcium-related GO terms in our study (calcium-mediated signaling, cellular response to calcium ion, positive regulation of cytosolic calcium ion concentration, and calcium ion binding) and the calcium signaling pathway were significantly enriched. They imply a role of calcium signaling in comb growth because calcium is an important material for bone deposition. In addition, the DEGs were found to enrich the GO terms including cell communication, cell migration, cell-cell signaling as well as the KEGG pathways of cytokine-cytokine receptor interactions, demonstrating that cytokine-mediated cellular interactions might be involved in the regulation of comb size.

By constructing a protein-protein interaction network for DEGs, we found a list of key genes: *STK32A*, *PI3KR1*, *EDN1*, *HSPA5*, and *HSPA8*. As the up- and down-regulated genes interacting with most genes of the network, serine/threonine kinase 32 A (*STK32A*) and phosphoinositide-3-kinase regulatory subunit 1 (*PIK3R1*) had a direct relationship according to STRING database. *STK32A* encodes a serine/threonine kinase, which involved numerous biological processes. In mice, the misexpression of *STK32A* is identified as a candidate fertility factor^38^. A previous study shows that a larger comb size could be used as an indicator of fecundity^7^, and up-regulation of *STK32A* in the BC group might promote comb growth. *PIK3R1* is associated with insulin resistance^39^. In our study, seven genes were enriched in the insulin resistance signaling pathway. It would be possible that *PIK3R1* regulates comb growth by interacting with the genes involved in the signaling pathway. Endothelin 1 (*EDN1*) is a potent vasoconstrictor mainly produced by endothelial cells, and it plays a role in restricting cartilage differentiation in Zebrafish^40^, suggesting that this gene has a similar function as *CHADL* in comb growth. Heat shock protein family A member 5 (*HSPA5*) and heat shock protein family A member 8 (*HSPA8*) encode two different proteins belonging to the heat shock protein 70 (HSPA) family, which is known for its role in responses to heat stress^41^. Considering the comb involvement in heat regulation, these genes *HSPA5* and *HSPA8* might participated in comb growth, but their involvement details are not clear.

The present study provides global transcriptome analysis in the comb tissues of chickens differing extreme comb size. The results suggested that the identified DEGs were related to the phenotypic differences of these two groups. The function enrichment analysis revealed that the DEGs were involved in cell communication and calcium signaling, and shed light on their potential regulation roles in chicken comb growth. Moreover, *BMP2*, *CHADL*, *STK32A*, *PIK3R1*, *EDN1*, *HSPA5*, and *HSPA8* might have great impacts on comb growth.

## Materials and Methods

### Ethics statement

All animal experiments were approved by the Animal Care and Use Committee at the Institute of Poultry Science, Chinese Academy of Agricultural Science (Approval ID: S20160605) and conducted at the institute. All of the experiments followed relevant guidelines and regulations set by the Ministry of Agriculture of the People’s Republic of China.

### Sampling

All roosters used in the present study were collected from a Partridge Shank chicken breeding line in Jiangsu Lihua Animal Husbandry Company (Jiangsu, China). All chickens lived under the same conditions and were raised using a standardized feeding method with free access to water. To collect roosters with different comb sizes for sequencing, the comb areas and heights of 100 nine-week-old male chickens were measured using a computer-assisted method. Based on the measurement results, three birds with the biggest comb sizes and three with the smallest phenotypes were selected for RNA-seq. The thresholds for the BC group were the comb area of not less than 2,450 mm^2^ and comb height of not less than 39 mm, while those for the SC group were the comb area of not greater than 680 mm^2^ and comb height of not greater than 21 mm. Details about the selected roosters are shown in Table 1.

**Table 1:** Comb areas and heights of six roosters involved in RNA-seq.

Sample	BC group				SC group			
	BC1	BC2	BC3	Means	SC1	SC2	SC3	Means
Comb area (mm^2^)	2836.0	2506.8	2486.4	2609.8	580.3	572.8	671.6	608.2
Comb height (mm)	45.1	46.0	39.3	43.5	18.6	18.2	20.5	19.1

BC: Big comb size; SC: Small comb size.

Comb tissues were harvested from the selected chickens after sacrificed by euthanasia, temporarily frozen in liquid nitrogen and stored at -80 °C until further manipulation.

### Total RNA extraction

Total comb RNA was extracted from each comb sample using TRIzol reagent (Invitrogen, USA) according to the manufacturer’s instructions. RNA concentration and integrity were estimated by NanoDrop 2000 (Thermo, USA) and Agilent 2100 Bioanalyzer (Agilent Technologies, USA), respectively.

### Library preparation for sequencing

TruSeq Stranded Total RNA with Ribo-Zero Gold kit (Illumina, USA) was used to establish strand-specific RNA-seq libraries following the manufacturer’s recommendations. In brief, Ribosomal RNA was removed from 3 μg RNA. After RNA fragmentation, double-stranded cDNA was synthesized by replacing dTTPs with dUTPs in reaction buffer used for second-strand cDNA synthesis. The resulting double-stranded cDNA was ligated to adaptors after being end-repaired and A-tailed. Single-stranded cDNA was then obtained using USER Enzyme, and PCR amplification was performed to enrich the cDNA libraries. Finally, PCR products were purified and the library quality was assessed on an Agilent 2100 Bioanalyzer system.

Sequencing was performed on an Illumina HiSeq. 2500 instrument to generate 150 bp paired-end reads using TruSeq PE Cluster Kit v3-cBot-HS (Illumina, USA).

### Sequencing data analysis

Quality control and read statistics were determined by FastQC (v0.11.2)^42^. After reads containing adapter or ploy-N and other low-quality reads were discarded, clean reads were aligned to the reference chicken genome (*Galgal 5.0*) using Tophat2 (v2.0.12)^20^. Transcripts were reconstructed and then compared with known transcripts to predict novel transcripts using Cufflinks software (v2.2.1)^20^; novel transcripts were performed with coding analysis using CPC software (v0.9)^21^. Gene structures were refined by comparing known transcripts with the reconstructed transcripts from the six transcriptome sequencing datasets using BLAST software.

### Identification of alternative splicing events and SNPs

AS events were detected using ASprofile software^22^ and were classified into 12 categories: alternative 5′ first exon (transcription start site, TSS), alternative 3′ last exon (transcription terminal site, TTS), skipped exon (SKIP), approximate SKIP (XSKIP), multi-exon SKIP (MSKIP), approximate MSKIP (XMSKIP), intron retention (IR), approximate IR (XIR), multi-IR (MIR), approximate MIR (XMIR), alternative exon end (AE), and approximate AE (XAE). SNP calling was performed using a genome analysis toolkit GATK2 (v3.2)^23^ based on the Unified Genotyper algorithm.

### Identification of differentially expressed genes

The expression level of each gene was calculated using HTSeq software (v0.6.1)^24^ and normalized with the RPKM method. DEGs between the BC and SC groups were analyzed using the edgeR R package^25^. *P*-value < 0.05 and |fold-change| > 1.5 were considered as significant thresholds.

### qPCR validation

To increase the reliability of this test, 30 comb samples were collected from the roosters aged 9 weeks in the same group including 15 big and 15 small comb size individuals, which were tested by qPCR. The details about the samples were provided in Supplementary Table S8. qPCR was performed on an ABI 7500 Real-Time PCR system (Applied Biosystems, USA) using KAPA SYBR Fast universal qPCR kit (Kapa Biosystems, USA). Specific primers of the genes shown in Supplementary Table S9 were designed using Primer Premier 5 and confirmed by Oligo 6.0. Using *β-actin* as a reference, relative expression levels of the genes were quantified using 2^(−ΔΔCt)^ methods^43^.

### Functional annotation and QTL location analysis of differentially expressed gene**s**

GO and KEGG pathway enrichment analysis of the DEGs was performed using DAVID online tool (https://david.ncifcrf.gov/, version 6.8)^26^. We performed QTL mapping for the DEGs by comparing the DEGs with chicken QTL chromosome positions using BEDTools^44^.

### Protein-protein interaction network analysis

Based on STRING database (http://string-db.org/, version 10.5)^44^, we analyzed the protein-protein interaction network for DEGs and further investigated the interaction between DEGs in the comb tissues of the BC and SC chickens. Cystoscope was used to visualize the protein-protein interaction network to find out key genes.

### Data availability

Raw and processed data in this study were deposited in the NCBI Gene Expression Omnibus (GEO, http://www.ncbi.nlm.nih.gov/geo) with the following accession number: GSE107815.

## Electronic supplementary material

Table S1

Table S2

Table S3

Table S4

Table S5

Table S6

Table S7

Table S8

Table S9
